# Plasmids in *Neisseria gonorrhoeae*: drivers of DoxyPEP failure and an emerging threat for current therapy

**DOI:** 10.1128/cmr.00252-25

**Published:** 2026-03-10

**Authors:** Tabea A. Elsener, Jo-Anne R. Dillon, William M. Shafer, Christoph M. Tang

**Affiliations:** 1 Sir William Dunn School of Pathology, University of Oxford 98972, Oxford, United Kingdom; 2 Department of Biochemistry, Microbiology and Immunology, College of Medicine and the Vaccine and Infectious Disease Organization, University of Saskatchewan 7235, Saskatoon, Saskatchewan, Canada; 3 Department of Microbiology and Immunology, Emory University School of Medicine, Veterans Affairs Medical Center, Decatur, Georgia, USA; Mayo Clinic Minnesota, Rochester, Minnesota, USA

**Keywords:** plasmids, DoxyPEP, gonococcus, p*bla*, pConj, *Neisseria gonorrhoeae*

## Abstract

Gonococcal disease is a serious threat to reproductive health, with cases and antimicrobial resistance steadily increasing. Currently, the β-lactam antibiotic ceftriaxone is the mainstay of treatment, while doxycycline post-exposure prophylaxis (DoxyPEP) is being implemented to reduce bacterial sexually transmitted infections. *Neisseria gonorrhoeae*, the causative agent of gonorrhoea, can harbor two resistance plasmids, p*bla* and pConj, which confer resistance to penicillin and tetracycline/doxycycline, respectively. Additionally, over 90% of isolates carry a small plasmid, pCryp, whose function remains unclear. Of concern, p*bla*-encoded β-lactamases only differ from extended-spectrum β-lactamases by one or two amino acid changes. DoxyPEP is completely ineffective against isolates carrying pConj expressing *tetM. tetM*-mediated resistance has increased in the gonococcus following introduction of DoxyPEP and is widespread in low- and middle-income countries. Therefore, plasmids pose a significant risk to the successful management of gonococcal infection. Here, we review current knowledge of the epidemiology and biology of gonococcal plasmids. We describe the hierarchy of acquisition and dependencies between plasmids in *N. gonorrhoeae* and their subsequent evolution, transfer dynamics, fitness costs, and benefits to provide a better understanding of the implications of the plasmids on public health interventions for controlling and treating gonococcal infection.

## INTRODUCTION

*Neisseria gonorrhoeae* (the gonococcus) is an obligate human pathogen that causes the sexually transmitted infection (STI) gonorrhoea (1). The gonococcus infects the mucosal surfaces of the urethra, cervix, pharynx, and eye. Most frequently, gonococcal infection results in cervicitis and urethritis (2); however, oropharyngeal and rectal infections are becoming increasingly common (3). Cervical infection is often asymptomatic but can lead to infection of the upper genital tract with serious complications such as infertility and pelvic inflammatory disease (4). In addition, gonorrhoea increases the risk of acquiring and spreading HIV (5, 6). There is no licensed vaccine available for gonorrhoea. However, an outer membrane vesicle-based serogroup B meningococcal (MenB) vaccine has shown cross-protective effects (vaccine effectiveness of ~30%) against gonorrhoea (7), and a gonorrhoea vaccination programme for high-risk groups has been launched in the United Kingdom (8). Still, the management of cases and their contacts depends almost entirely on effective diagnosis and antimicrobial treatment. Currently, the most widely recommended treatment for gonococcal infection is a single dose of the β-lactam antibiotic, ceftriaxone (9, 10). However, *N. gonorrhoeae* has evolved resistance against every antibiotic that has been used for the treatment of gonorrhoea (11), leading to its designation as a priority pathogen for the development of novel therapeutics and/or vaccines by the World Health Organization (12). For prevention, doxycycline, a tetracycline antibiotic, has been evaluated for post-exposure prophylaxis (DoxyPEP) as a public health measure against bacterial STIs among high-risk groups (13, 14). The use of a single agent taken on one occasion to prevent STIs caused by multiple pathogens (i.e., Chlamydia, *Treponema pallidum*, and *N. gonorrhoeae*) contrasts with HIV PEP in which three agents against a single pathogen are taken for 28 days. The spread of resistance is a major concern for DoxyPEP, and the prevalence of doxycycline resistance underlies some of its variable effects against gonococcal infection (15–17). Unsurprisingly, DoxyPEP was entirely ineffective against *N. gonorrhoeae* in Kenya (15), where gonococcal resistance to doxycycline is virtually ubiquitous (18).

Considerable attention has been paid to chromosomal antibiotic resistance in the gonococcus. The bacterium is naturally competent for DNA uptake, and acquisition of genetic material from non-invasive *Neisseria* spp. has led to the emergence of chromosomal resistance determinants, such as mosaic *penA* alleles, that confer resistance to cephalosporins including ceftriaxone (19, 20). Furthermore, point mutations in the 23 rRNA have arisen in distinct lineages of the gonococcus, leading to resistance against azithromycin (21). In contrast, there has been less focus on plasmid-mediated resistance, even though *N. gonorrhoeae* can harbor two plasmids which confer antimicrobial resistance (AMR) (22). Indeed, the acquisition and spread of p*bla* and pConj containing the *tetM* determinant in the second half of the 20th century contributed to the discontinuation of penicillin and tetracycline for treating gonorrhoea, respectively (23–25).

However, plasmid-mediated resistance is in the spotlight once again as the success of DoxyPEP against gonococcal infection depends on the prevalence of strains with pConj/*tetM*. For example, it is becoming appreciated that tetracycline/doxycycline resistance due to pConj bearing *tetM* is virtually ubiquitous in sub-Saharan Africa (where disease incidence is high) (18, 26, 27), limiting the utility of syndromic treatment with doxycycline and negating the increasingly popular public health intervention of DoxyPEP. Additionally, while reducing syphilis and chlamydia infections, in regions where DoxyPEP has been implemented, there has been a surge of cases caused by strains with plasmid-mediated resistance (28). Importantly, selection for plasmid-mediated resistance not only enhances the prevalence of plasmids themselves but will also drive the proliferation of highly resistant gonococcal strains that harbor them, amplifying the threat to current therapies. In addition, the p*bla*-encoded TEM β-lactamases only differ by one or two amino acids from extended-spectrum β-lactamases (ESBLs) such as TEM-43 (29), potentially threatening treatment with third-generation cephalosporins. Therefore, it is highly timely to reappraise current knowledge of plasmids in the gonococcus.

pConj and p*bla*, along with a third plasmid pCryp (also referred to as pJD1 (30), constitute the limited repertoire of plasmids in the gonococcus. Interestingly, these plasmids are also largely restricted *to N. gonorrhoeae*, so they are host-adapted. Here, we review current knowledge about the origin and molecular epidemiology of these narrow host range plasmids. We examine the basis for their long-term relationship with the gonococcus, and in particular, with certain lineages, and discuss their potential to undermine current approaches to treat and prevent gonococcal disease.

## THE GONOCOCCUS HARBOURS UP TO THREE PLASMIDS

**Fig 1:**
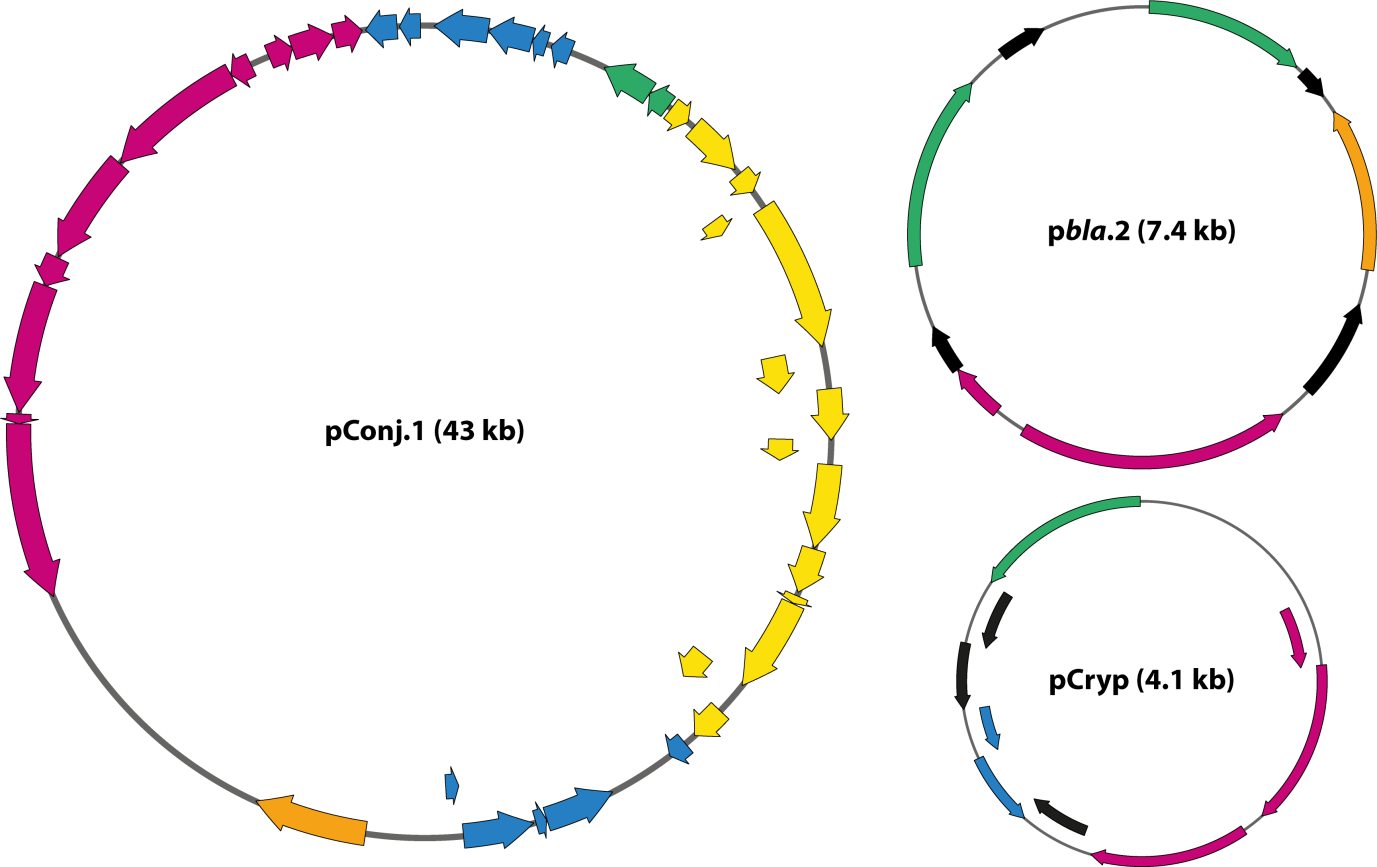
Plasmid maps of pConj, p*bla*, and pCryp with ORFs colored according to gene functions: orange, AMR; green, plasmid replication; pink, plasmid transfer; yellow, T4SS; blue, plasmid maintenance; black, other gene function.

Until recently, the epidemiology and evolution of plasmid-mediated resistance have remained poorly understood and, so far, have not been integrated into risk assessments for public health interventions or even mentioned in reports of increased doxycycline resistance following the introduction of DoxyPEP (28). Many bacteria carry a wide range of plasmids, which are important drivers of AMR (31–33). In contrast, only three plasmids have been described in *N. gonorrhoeae* (22), although there are variants of these plasmids (26, 34). Based on publicly available databases (e.g., PubMLST [35]), interrogation of genome sequence data from isolates obtained globally provides important information about the distribution of plasmids in *N. gonorrhoeae*. There are genome sequences for >15,000 gonococcal isolates from over 66 countries (https://pubmlst.org/). Although certain regions and countries are over-represented in these collections, they provide a valuable resource for understanding gonococcal population biology, AMR, and plasmids. Over 90% of gonococci carry the highly conserved 4.2 kb cryptic plasmid, pCryp (36–38). This plasmid has ten open reading frames (ORFs), which encode a replicase, a VapDX toxin-antitoxin (TA) system, mobilization proteins, and four proteins of unknown function (26) (Fig. 1). Despite its prevalence, little is known about the function or origin of pCryp, even though it is found in the earliest sequenced gonococcal isolate, which was recovered in 1928 (39).

pConj (39–42 kb, Fig. 1) is present in around a third of gonococci (26). This plasmid encodes a Type IV secretion system (T4SS) and can transfer itself between gonococci by conjugation. The genomic organization of pConj has been comprehensively described previously (40), but more recent analysis shows the plasmid can be grouped into seven variants (26). pConj variants 1 to 4 carry *tetM*, conferring resistance to tetracycline antibiotics including doxycycline, so limit the efficacy of DoxyPEP; pConj 1 and 2 carry the *tetM* allele 2 (also referred to as “American”), while pConj 3 and 4 carry allele 1 (“Dutch”) (26). Variants 5 to 7 lack *tetM* and are referred to as markerless. pConj variants also differ in their repertoire of TA systems and components of the T4SS (26). This can affect the transfer and distribution of pConj. For example, pConj.2 contains a version of the T4SS protein TrbL that reduces pConj conjugation (38), with pConj.2 restricted to a few lineages in the gonococcal population (26).

The β-lactamase plasmid p*bla* carries a truncated Tn*2* transposon encoding a TEM β-lactamase conferring resistance to penicillin and ampicillin (41). p*bla* cannot transfer itself, but it encodes a relaxase (MobA) and contains an *oriT*, so p*bla* is mobilizable by pConj (42).

While at least seven variants of p*bla* have been described (41, 43, 44), three of them accounted for more than 99% of typeable p*bla* sequences in a global collection of over 15,000 isolates (34). The prototypic p*bla*.2 (p*bla* Asia, 7.4 kb) contains nine ORFs, which are derived from Tn*2* or involved in plasmid replication or mobilization (Fig. 1). Unusually for such a small plasmid, p*bla*.2 carries three origins of replication and encodes two replication initiation proteins; RepA and RepB initiate replication from *ori*1 and *ori*2/3, respectively (45). p*bla*.2 preferably initiates replication from *ori*2/3 in *Escherichia coli*, and plasmids replicating from *ori*1 differ from *ori*2/3 replicating plasmid variants in their host range, plasmid incompatibility, and their dependency on host DNA polymerase I for their replication (45). In contrast, p*bla*.1 (p*bla* Africa, 5.6 kb) is 1.4 kb smaller than p*bla*.2 and only encodes RepA, while p*bla*.3 (5.1 kb, p*bla* Rio/Toronto) lacks the mobilization region (34, 41).

The p*bla* variants are also associated with distinct TEM variants (Table 1) (34). p*bla*.1 mostly carries TEM-1, with a subpopulation of p*bla*.1 associated with a P14S substitution in the leader peptide of TEM. p*bla*.2 expresses TEM-1 or TEM-135, while p*bla*.3 exclusively carries TEM-135, which harbors an M182T substitution relative to TEM-1. The M182T substitution is a stepping stone mutation toward an ESBL as it increases the stability of the enzyme, compensating for destabilizing effects of amino acid changes in the active site in ESBLs (46). While in *E. coli*, the M182T substitution in TEM-135 reduces the penicillin minimum inhibitory concentration (MIC) (46), TEM-135 increases resistance in *N. gonorrhoeae* (45), likely conferring an advantage to strains containing p*bla*.3 and expressing this β-lactamase. While the basis for this difference is unknown, the presence of TEM-135 is associated with increased levels of cellular β-lactamase (shown by Western blot analysis) without a change in mRNA levels (45). This suggests that the effect of TEM-135 is post-transcriptional, with a potential explanation being that *E. coli* and the gonococcus use distinct mechanisms to process periplasmic β-lactamases. Given the importance of TEM-135, further studies are warranted to define the mechanism by which this β-lactamase confers higher MICs. All p*bla* encode TEM β-lactamases, but a p*bla*-carrying isolate with reduced penicillin resistance due to a 6 bp deletion in *bla*TEM has been reported (47).

**TABLE 1:** Characteristics of the three major p*bla* variants.

p*bla* variant	Size	Defining gene absence	Associated TEM	Mobility
p*bla*.1/p*bla* Africa	5.6 kb	*repB*	TEM-1 or TEM-1_P14S_	Mobilizable
p*bla*.2/p*bla* Asia	7.4 kb	–^*a*^	TEM-1 or TEM-135	Mobilizable
p*bla*.3/p*bla* Rio/Toronto	5.1 kb	*mobA*, *mobC*	TEM-135	Non-mobile

^*a*^ – indicates gene absence that is reported with respect to p*bla*.2, which is the largest of the three major plasmid variants.

## GONOCOCCAL PLASMIDS WERE ACQUIRED FROM RELATED PATHOGENS

Similar to the biology of pCryp, the origin of this plasmid remains enigmatic. However, pCryp shares a long and close evolutionary history with *N. gonorrhoeae*, (35) with it being prevalent in and mostly limited to the gonococcus (38).

The earliest pConj carrying *N. gonorrhoeae* isolate dates back to 1940 and harbors a markerless version of the plasmid (39). However, due to the limited number of isolates predating this, the plasmid might have been circulating in the gonococcus before then. pConj is also sporadically found in *Neisseria meningitidis* (present in around 0.5% meningococci) (38). Despite this, the plasmid is more genetically diverse in the meningococcus compared to the gonococcus, indicating that pConj has a much longer evolutionary history with *N. meningitidis* compared to *N. gonorrhoeae* (38). Further phylogenetic analysis suggests the plasmid was transferred from the meningococcus into *N. gonorrhoeae* on at least two occasions. The first introduction of pConj into *N. gonorrhoeae* was followed by clonal expansion and dissemination in the population; another introduced variant, pConj.2, has greatly reduced mobility and is only found in a limited range of gonococcal isolates (38).

The first *tetM*^+^ pConj carrying gonococcal isolates associated with high-level resistance to tetracycline, doxycycline, and minocycline was described in 1983 (48). While chromosomal mutations in genes such as *mtr* and *porB* can confer low-level tetracycline resistance (MICs of <4 µg/mL), the acquisition of *tetM* results in high-level resistance to tetracycline (MIC > 16 µg/mL), doxycycline (MICs 8 to 24 μg/mL), and minocycline (MICs of 12 to 32 μg/mL) (49). An early study reported *tetM*-carrying isolates in both heterosexual and MSM networks, with isolates belonging to distinct auxotrophs, consistent with widespread plasmid-mediated dissemination of *tetM* (49). Before its description in the gonococcus, *tetM*-mediated tetracycline resistance had been reported in genitourinary tract organisms such as *Mycoplasma hominis* (50), *Ureaplasma urealyticum* (51), and *Gardnerella vaginalis* (52). However, in all these organisms, *tetM* is on the chromosome. Subsequently, it was shown that *tetM* on pConj is derived from Tn*916* with a deletion of a large section of the transposon, ensuring its stable association with the plasmid (53). Furthermore, there are two distinct *tetM* alleles on pConj (referred to as Dutch and American *tetM*), which suggests that pConj acquired this resistance determinant on at least two occasions (54).

Plasmid-mediated penicillin resistance in *N. gonorrhoeae* was first reported in 1976 in the United Kingdom in a strain epidemiologically linked to West Africa (55). In the same year, p*bla*.2 was reported in a variety of strains in North America with epidemiological links to the Far East (56). Both p*bla* variants contain a truncated version of the transposon Tn*2* encoding a TEM β-lactamase. Related small β-lactamase-producing plasmids had been reported in *Haemophilus* spp. (57–59). Analysis of the distribution of Tn*2* and genes in the backbone of p*bla* in *Haemophilus* sequences on the public database PubMLST (35) showed that, while in *Haemophilus influenzae*, Tn*2* and p*bla* sequences rarely co-occur in the same isolate, in the STI pathogen *Haemophilus ducreyi*, around a quarter of isolates harbor TEM-1-carrying p*bla* (45). Of note, two distinct plasmid variants (9.1 and 10.9 kb in size) that are almost identical to gonococcal p*bla*.1 and p*bla*.2, respectively, have now been identified in *H. ducreyi* (45). The major difference between the plasmids in *H. ducreyi* and *N. gonorrhoeae* is that Tn*2* has been truncated in the latter. This suggests that p*bla* was originally in *H. ducreyi*; subsequent introduction into the gonococcus was associated with deletion of part of Tn*2* while leaving the *bla*TEM gene intact (45, 60, 61).

In summary, while chromosomal resistance determinants in *N. gonorrhoeae* have mostly arisen from the transfer of genetic material from commensal *Neisseria* spp. (11), plasmids mediating AMR have been shared between pathogens. Furthermore, although *tetM* and *bla*TEM are present on transposons, both transposons are truncated in the gonococcus, confining the resistance genes to the plasmids.

## PLASMIDS ARE UNEVENLY DISTRIBUTED IN THE GONOCOCCAL POPULATION

A major challenge for plasmid biology is assembling intact plasmid sequences from whole-genome short-read sequences of bacterial isolates. Until recently, gonococcal plasmid variants were identified by PCR (62, 63), so analysis of plasmid distribution has been limited to discrete collections. However, the ability to resolve the gonococcal population structure by core genome clustering (64), combined with our implementation of typing schemes for pConj (26) and p*bla* (34), now enables the use of publicly available whole genome sequences to study the spread of these plasmids in gonococci. Consequently, recent analyses have provided an unprecedented view of the population biology of these host-restricted plasmids (26, 34).

**Fig 2:**
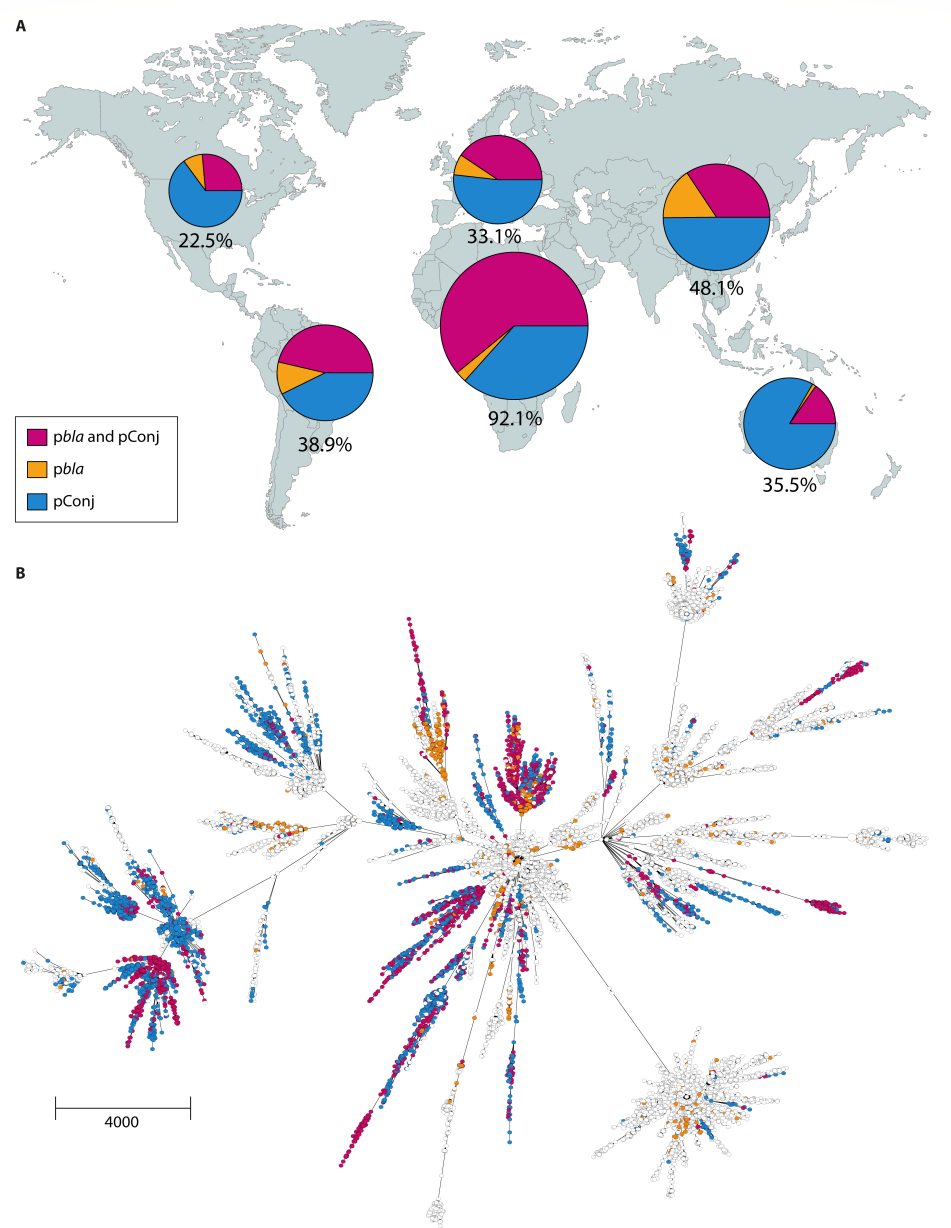
Epidemiology of the gonococcal resistance plasmids. (**A**) World map with plasmid prevalence in different geographical areas. Pie charts are scaled according to plasmid prevalence in a specific geographic region and show the proportion of p*bla*, pConj, or both plasmids occurring together in plasmid-carrying isolates. (**B**) Minimum spanning tree of gonococcal isolates clustered according to core genomic allelic differences. Individual isolates are represented as dots, which are colored according to plasmid carriage. The figures were generated using the data set from reference 34, accessible at https://doi.org/10.6084/m9.figshare.23638992.v1, which includes 15,532 isolates from 66 countries, including all six WHO regions, and spans the years 1928 to 2022*.*

Approximately 10% of *N. gonorrhoeae* isolates harbor all three gonococcal plasmids (397 of 3,724 isolates) with pConj present in about one-third (1,089 of 3,724 isolates) (26) and p*bla* in around one in five of sequenced gonococci (2,758 of 15,529 isolates) (34); pCryp is almost ubiquitous (3,513 of 3,724) (26). Of note, p*bla* is highly associated with pConj, with over 90% of strains with p*bla* also containing pConj (pConj in 399 of 441 isolates with p*bla*) (26). Furthermore, the prevalence of gonococcal resistance plasmids differs across the world (Fig. 2A). Over 90% of isolates from Africa, which has the highest burden of disease (27), contain pConj +/− p*bla* (18, 26, 34). Indeed, there is a negative correlation between plasmid-mediated resistance in the gonococcus and a country’s gross domestic product (GDP) (26); this association is not seen with markerless pConj, indicating that resistance conferred by the plasmids is responsible for this association.

p*bla* variants are also linked with different geographic areas; p*bla*.2 is largely found in isolates from Asia, while p*bla*.3 is more prevalent in high-income countries (34). In contrast, p*bla*.1 is the most frequent variant and found globally.

In addition to their geographic distribution across different countries and regions, gonococcal plasmids are also associated with specific bacterial populations, reflecting host-range preferences and evolutionary relationships. *N. gonorrhoeae* has a non-clonal population structure due to frequent horizontal gene transfer (HGT) between isolates (65). To resolve the population structure, gonococcal isolates can be clustered from whole genome sequence data according to 1,668 loci that form its core genome (i.e., present in >95% of isolates), generating core genome clusters (Ng_cgc) (64). Both p*bla* and pConj are associated with distinct Ng_cgcs (Fig. 2B) (26, 64), suggesting that certain plasmid-strain combinations are favorable. This is emphasized by the observation that Ng_cgcs with high levels of plasmid carriage do so whether they are found in high- or low-income countries (34). The basis for this is currently unknown.

## PLASMID TRANSFER BETWEEN GONOCOCCAL STRAINS

pConj is conjugative and can transfer itself between isolates as it encodes a T4SS, a relaxase, and contains an origin of transfer (*oriT*). The relaxase nicks the plasmid at the *oriT* and guides single-stranded plasmid DNA through the T4SS, allowing contact-dependent plasmid transfer (66). There have been a wide range of estimates of the rate of pConj transfer (67, 68), potentially reflecting differences in experimental conditions, donor/recipient strains, or pConj variants. However, in isogenic matings, pConj variants 1, 3, and 4 transfer at remarkably high rates, with ~80% of recipients acquiring the plasmid after 4 h of contact with a donor (38, 45). These are among the highest transfer rates of any naturally occurring plasmid, indicating constitutive expression of the conjugation machinery (42). In contrast, pConj.2 and 7 have reduced transfer ability (38, 45), which is reflected by the limited distribution of these plasmid variants in the gonococcal population, compared to highly transferable pConj variants (26).

p*bla* also encodes a relaxase and *oriT* (34, 69) but depends on the T4SS encoded by a co-resident conjugative plasmid to facilitate its transfer. In the laboratory, p*bla* can be transferred by different IncP conjugative plasmids (20, 70). However, in *N. gonorrhoeae*, which lacks other conjugative plasmids, p*bla* transfer depends on pConj, and p*bla* mostly co-occurs with pConj in isolates (26). Of note, p*bla* is mostly found in association with highly mobile pConj variants (45), indicating that the success of p*bla* depends on its relationship with pConj. In contrast to p*bla* variants 1 and 2, p*bla*.3 lacks the mobilization region (34, 69), rendering this variant immobile (45). Other modes of transfer, such as co-integration into a helper plasmid (71, 72), encapsulation into outer membrane vesicles (OMVs) (73), or natural transformation (74), have been reported for p*bla*. However, the absence of homologous sequences in p*bla* and pConj and the lack of DNA uptake sequences (DUS), required for efficient transformation in *N. gonorrhoeae* (75), in p*bla* limit non-conjugal transfer. Consequently, p*bla*.3 is limited in the gonococcal population to a few closely related lineages.

## MAINTENANCE AND EVOLUTION OF PLASMIDS IN THE GONOCOCCAL POPULATION

Although tetracycline and penicillin are no longer recommended for treating gonorrhoea, pConj and p*bla* have remained prevalent in the gonococcal population (39, 45), suggesting that these selfish genetic elements are stably inherited by the bacterium. Plasmids can deploy numerous mechanisms to ensure they are not lost by bacteria in the absence of selection (76, 77). For large plasmids, these mechanisms include partitioning systems that separate plasmids to the poles of bacteria before cell division (78) and TA systems. TA systems promote plasmid maintenance through post-segregational killing, in which daughter cells that fail to inherit a plasmid are killed through the action of a stable toxin once its unstable cognate antitoxin has been degraded (79). In contrast, small plasmids frequently rely on a high copy number to minimize loss after division (80, 81). pCryp encodes a VapDX TA system, which is also found in *Helicobacter pylori* (82) and *H. influenzae* (83).

pConj encodes a ParAB partitioning system, two epsilon-zeta type II TA systems, as well as an orphan VapD toxin (40), and pConj shows remarkably little loss in the laboratory even after >120 generations (39, 40). Deletion of *parB* was reported to lead to plasmid loss rates of 15% after only a few generations, which was further accelerated by the additional removal of the TA systems, resulting in complete loss of pConj (39). The pConj VapD toxin can be neutralized by the VapX antitoxin encoded by pCryp (39), indicating a functional dependency of pConj on the pCryp TA system, and a hierarchy between the two plasmids. However, removal of individual TA systems resulted in minimal loss, highlighting the redundancy of TA systems in maintaining pConj.

Interestingly, a decrease in plasmid-mediated penicillin and tetracycline resistance was noted in Hong Kong upon the emergence of quinolone resistance (84). Quinolones can affect plasmid maintenance as they target DNA gyrase and topoisomerase, which are involved in plasmid segregation (85), and ciprofloxacin can enhance pConj loss in the absence of TA systems (39). Furthermore, a recent study revealed that a *Pseudomonas aeruginosa*-derived quinolone N-oxide antibiotic selectively targets pConj-carrying *N. gonorrhoeae* by triggering the release of the zeta1 toxin, leading to bacterial death (86).

No plasmid maintenance systems have been described for p*bla*. However, the plasmid encodes a putative Fic antitoxin (NEIS2960) with a potential associated toxin encoded on the gonococcal chromosome (34). It remains to be determined whether NEIS2960 contributes to p*bla* maintenance in gonococci. p*bla* copy number ranges between 1 and >6 copies per chromosome (45). Even considering the polyploid nature of the gonococcus (with approximately six chromosome copies per diplococcus (87)), p*bla* copy number alone cannot explain its stability within gonococci. However, p*bla* segregational loss could be augmented by sporadic antibiotic selection and HGT of p*bla*.

p*bla* variants differ in their copy number, with an increased copy number of p*bla*.2 (6 copies/chromosome in contrast to 1–2 copies of p*bla*.1 and 3), which is associated with a significant fitness cost for this variant and a competitive disadvantage of p*bla*.2-carrying isolates (45). Differences in fitness costs are also reflected at a population level, with p*bla*.2 replaced by p*bla*.1 carrying isolates in Guangdong, China, a region previously dominated by p*bla*.2-carrying isolates (88). Overall, the fitness cost of p*bla*.2 is reflected in a low plasmid prevalence of p*bla*.2-associated lineages and a decrease of this plasmid variant over time in the global gonococcal population (45). In contrast, p*bla* variants 1 and 3 remain stable over time, potentially due to their lack of fitness costs and the increased resistance levels provided by p*bla*.3 and the mobility of p*bla*.1.

The minimal fitness cost of p*bla*.1 and 3 and the effective maintenance systems of pConj indicate that there might be no going back. Discontinuation of antibiotics has not reduced plasmid carriage in gonococci, so antibiotic cycling is likely to be unhelpful in controlling plasmid-mediated resistance in *N. gonorrhoeae*.

## PERSPECTIVES

High levels of AMR endanger the future of gonococcal treatment and control (1, 11). The emergence of chromosomally encoded resistance to penicillin over nearly 40 years has involved an accumulation of mutations in genes encoding the penicillin binding proteins PBP2 (PenA) and PBP1 (PonA), the MtrCDE multidrug efflux pump, and the outer membrane porin PorB (24); carriage of mosaic *penA* (encodes PBP2) comes at a cost for the bacterium, which can be mitigated by compensatory mutations (89). In contrast, HGT of resistance provides a quantum leap in the development of resistance, with no associated fitness costs for several gonococcal resistance plasmids, such as p*bla*.1 and 3, and pConj.1 (39, 45), which are found in diverse lineages across the gonococcal population (Fig. 2B).

Given its prevalence, future work should be directed to understand whether, and if so how, pCryp contributes to gonococcal biology; it is possible that this small plasmid is merely a selfish entity, which exploits its host without offering benefits. While p*bla*-encoded TEM β-lactamases do not currently confer resistance to third-generation cephalosporins, TEM-135 has evolved in the gonococcus and is prevalent in p*bla*.3, which is associated with lineages that are prevalent in high-income countries. TEM-135 confers increased penicillin resistance and is a stepping stone toward an ESBL while enhancing resistance to penicillin (29); the threat this poses to current therapies is obvious. The increased prevalence of pConj in the gonococcal population, probably through the indiscriminate use of doxycycline, will not only increase the spread and carriage of the highly transmissible conjugative plasmid but also select for proliferation of p*bla*. Together, the spread of pConj and TEM-135-carrying p*bla* exemplifies the evolutionary dynamics that could drive the success of p*bla* and the emergence of strains with transmissible resistance that undermine current therapies.

While DoxyPEP has successfully reduced the incidence of syphilis and chlamydia in high-risk populations (16, 17), its effect on gonorrhoea is mixed at best, with the outcome dependent on the local prevalence of pConj. Of note, the failure of DoxyPEP in preventing gonococcal disease among cisgender women in Kenya (15) was predictable at the outset, given the high prevalence of pConj in this country (18). Also, doxycycline has been recommended for the syndromic treatment of STIs in low-resource settings (18, 26, 34), potentially explaining the high plasmid prevalence in low-income countries. Therefore, while DoxyPEP could temporarily reduce cases in high-income settings (90), where pConj prevalence is low, this will not last. *tetM* is spread by a highly mobile plasmid with limited fitness costs, so the plasmid might appear in novel lineages, while those lineages which already harbor pConj are likely to proliferate. This is a significant concern for lineages that carry both chromosomal resistance against third-generation cephalosporins and pConj/*tetM* (91, 92). By way of comparison, HIV PEP uses three-drug regimen directed at a single pathogen over 28 days to maximize efficacy while minimizing the risk of resistance. In contrast, DoxyPEP employs the single dose of a single agent against multiple pathogens; the broad-spectrum activity of doxycycline can disrupt the microbiome and carries the concomitant risk of selecting for resistant strains, especially pConj-carrying gonococci, and co-selecting additional resistance determinants through bystander effects (28). Going forward, any consideration of the long- and short-term risks vs benefits of public health interventions must factor in whether the relevant resistance is plasmid or chromosomally mediated in the gonococcus, and active surveillance implemented to examine the impact of DoxyPEP, a partially effective public health measure, on the evolution of this important human pathogen.
